# Co-evolutionary Signals Identify *Burkholderia pseudomallei* Survival Strategies in a Hostile Environment

**DOI:** 10.1093/molbev/msab306

**Published:** 2021-10-18

**Authors:** Claire Chewapreecha, Johan Pensar, Supaksorn Chattagul, Maiju Pesonen, Apiwat Sangphukieo, Phumrapee Boonklang, Chotima Potisap, Sirikamon Koosakulnirand, Edward J Feil, Susanna Dunachie, Narisara Chantratita, Direk Limmathurotsakul, Sharon J Peacock, Nick P J Day, Julian Parkhill, Nicholas R Thomson, Rasana W Sermswan, Jukka Corander

**Affiliations:** 1 Mahidol-Oxford Tropical Medicine Research Unit, Faculty of Tropical Medicine, Mahidol University, Bangkok, Thailand; 2 Parasites and Microbes Programme, Wellcome Sanger Insitute, Hinxton, United Kingdom; 3 Bioinformatics & Systems Biology Program, King Mongkut’s University of Technology Thonburi, Bangkok, Thailand; 4 Department of Mathematics, University of Oslo, Oslo, Norway; 5 Department of Mathematics and Statistics, Helsinki Institute of Information Technology, University of Helsinki, Helsinki, Finland; 6 Melioidosis Research Center, Khon Kaen University, Khon Kaen, Thailand; 7 Department of Biochemistry, Faculty of Medicine, Khon Kaen University, Khon Kaen, Thailand; 8 Oslo Centre for Biostatistics and Epidemiology, Oslo University Hospital, Oslo, Norway; 9 Department of Microbiology and Immunology, Faculty of Tropical Medicine, Mahidol University, Bangkok, Thailand; 10 Department of Biology and Biochemistry, The Milner Centre for Evolution, University of Bath, Bath, United Kingdom; 11 Centre for Tropical Medicine and Global Health, University of Oxford, Oxford, United Kingdom; 12 Department of Medicine, University of Cambridge, Cambridge, United Kingdom; 13 Department of Veterinary Medicine, University of Cambridge, Cambridge, United Kingdom; 14 Department of Biostatistics, University of Oslo, Oslo, Norway

**Keywords:** *Burkholderia pseudomallei*, co-selection study, nutrient depletion

## Abstract

The soil bacterium *Burkholderia pseudomallei* is the causative agent of melioidosis and a significant cause of human morbidity and mortality in many tropical and subtropical countries. The species notoriously survives harsh environmental conditions but the genetic architecture for these adaptations remains unclear. Here we employed a powerful combination of genome-wide epistasis and co-selection studies (2,011 genomes), condition-wide transcriptome analyses (82 diverse conditions), and a gene knockout assay to uncover signals of “co-selection”—that is a combination of genetic markers that have been repeatedly selected together through *B. pseudomallei* evolution. These enabled us to identify 13,061 mutation pairs under co-selection in distinct genes and noncoding RNA. Genes under co-selection displayed marked expression correlation when *B. pseudomallei* was subjected to physical stress conditions, highlighting the conditions as one of the major evolutionary driving forces for this bacterium. We identified a putative adhesin (*BPSL1661*) as a hub of co-selection signals, experimentally confirmed a *BPSL1661* role under nutrient deprivation, and explored the functional basis of co-selection gene network surrounding *BPSL1661* in facilitating the bacterial survival under nutrient depletion. Our findings suggest that nutrient-limited conditions have been the common selection pressure acting on this species, and allelic variation of *BPSL1661* may have promoted *B. pseudomallei* survival during harsh environmental conditions by facilitating bacterial adherence to different surfaces, cells, or living hosts.

## Introduction

One of a long-standing research question in evolutionary biology is to understand how natural selection operates across the population. Common selective pressures likely result in consistent patterns across individuals regardless of the background population. An unbiased genome-wide scan for cooccurrence of genetic markers such as single-nucleotide polymorphisms (SNPs), indels, orthologous genes, or pathways can be used to identify functional patterns caused by shared selective pressures. Assuming a perfect linkage equilibrium where genetic markers are inherited independently and combined at random, a nonrandom cooccurrence of these markers likely suggests their interactions that confer a selective advantage and thus are co-selected together. Depending on types of selection, the frequency of co-selected markers may coincrease until they reach fixation (positive selection) or comaintain at low frequency with multiple alleles at selected sites (balancing selection). However, linkage equilibrium is rare with genetic markers often inherited together forming linkage disequilibrium (LD) blocks. These physical blocks can be broken down by recombination events, thereby enabling an opportunity to investigate selective pressures through co-selected markers when LD structure is carefully considered. Although recombination in bacteria does not occur at every generation, some species such as *Campylobacter jejuni*, *Helicobacter pylori*, *Neisseria gonorrhoeae*, *Streptococcus pneumoniae*, *Vibrio parahaemolyticus*, and *Burkholderia* species frequently recombine resulting in disruptions of LD blocks (Nandi et al. 2015; Arnold et al. 2018; Cui et al. 2020; Zhou et al. 2020). Identifications of co-selected genetic markers among these recombinogenic species have shed light on groups of interacting genes required for the species adaptation including antimicrobial resistance genes in host-restricted pathogens or genes essential for cell integrity and mobility in an environmental bacterium (Pensar et al. 2019; Schubert et al. 2019; Cui et al. 2020). These studies motivated us to search for patterns of co-selection in a recombinogenic bacterium, *Burkholderia pseudomallei* to investigate the selection pressures that have acted on this species, and key genes that allowed its adaptation. 

The environmental bacterium *B. pseudomallei* has been increasingly recognized as an emerging human pathogen and a cause of melioidosis, a rapidly fatal infectious disease that annually affects and kills an estimated number of 165,000 and 89,000 patients, respectively (Limmathurotsakul et al. 2016). The bacterium can be isolated from the soil in many tropical and subtropical regions. It is most abundant at depths ≥ 10 cm from the surface but can move from deeper soil layers to the soil surface during the rainy season and multiply; this has been linked with an increase in disease incidence after heavy rainfalls (Inglis and Sagripanti 2006; Wiersinga et al. 2018). The presence of *B. pseudomallei* has been associated with nutrient-depleted soil and soil modified by long-term human activity (Coenye and Vandamme 2003; Limmathurotsakul et al. 2013; Baker et al. 2015; Ngamsang et al. 2015; Hantrakun et al. 2016; Manivanh et al. 2017); thereby connecting human manipulation of soil physicochemistry that promotes the abundance of this species. *Burkholderiapseudomallei* can survive extreme environmental conditions ranging from dry terrain in deserts (Yip et al. 2015) to distilled water (Pumpuang et al. 2011) (no nutrients); the latter ongoing experiment has been run for over 25 years. Such diverse and extreme environmental conditions likely impose unique selection pressures on *B. pseudomallei* but the key genetic factors that mediate these adaptations are unknown.

We applied a hypothesis-free co-selection analysis, known as genome-wide epistasis and co-selection study (GWES) (Pensar et al. 2019; Schubert et al. 2019; Cui et al. 2020) to detect co-selection signals in a collection of 1,136 *B. pseudomallei* isolates from 1935 to 2013 and validate our findings on a more recent collection of 875 isolates from 1976 to 2018 (supplementary fig. S1 and table S1, Supplementary Material online), enabling us to determine a set of SNPs under selective pressure. To explore the function of the co-selection signals, SNPs were mapped to genes and noncoding RNA (ncRNA) of *B. pseudomallei* reference genome K96243 resulting in co-selected gene–gene pairs, gene–nc RNA pairs, and ncRNA–ncRNA pairs. Using condition-wide gene expression data (Ooi et al. 2013; Mohd-Padil et al. 2017), we next searched for conditions at which co-selected gene–gene pairs displayed gene expression correlations, which likely reflect selective conditions *B. pseudomallei* has been subjected to. Finally, we used gene knockout assays to confirm the function of a gene under co-selection hotspot as well as the conditions that likely shaped *B. pseudomalle*i evolution.

## Results and Discussion

### Detection of Co-selection Signals

**Fig. 1.**
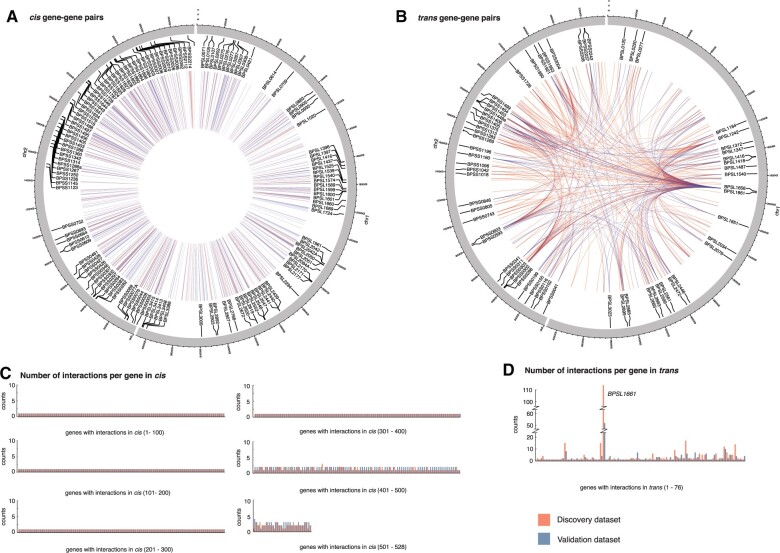
Co-selected gene–gene pairs in discovery and validation data sets. Circos diagrams showing co-selected gene–gene pairs in *cis* (*A*) and *trans* (*B*). Any pairs located within 7.68 kb (95th percentile of transcription fragments) were categorized as *cis*, whereas those located further apart were grouped as *trans*. Bar charts summarizing the number of interacting partners per gene for *cis* (*C*) and *trans* (*D*) linkage. Data from discovery and validation data sets were highlighted in red and blue, respectively.

Our search for co-selection signals in the discovery data set resulted in 13,061 SNP–SNP pairs spanning chromosome I (5,550 pairs), chromosome II (7,309 pairs), and between chromosomes I and II (202 pairs); of which 8,035 pairs (61.5%) could be replicated in the validation data set (see Materials and Methods). Although the unique pairs observed in each data set could be due to different sampling timeframe or linkage structure, the congruence of the co-selection signals in the discovery and validation data sets (supplementary table S2, Supplementary Material online) demonstrates that these complex patterns are consistent through time. The co-selected SNPs spanned genes, ncRNA, and intergenic regions, each of which accounted for 69.5%, 6.8%, and 23.7%, respectively in the discovery data set, with similar ratio observed in the validation data set. Most of SNP–SNP pairs were located either within the same gene or in close physical proximity (supplementary fig. S2 and table S2, Supplementary Material online); an observation driven by LD. These pair were separated by small physical distances and are unlikely to have had recombination events between them, resulting in so-called LD-mediated links. Although pairs located in close proximity are most likely explained by LD structure, some of them may represent genuine *cis* interacting partners. These may include SNPs in regulatory regions upstream of genes, such as DNA binding sites for RNA polymerase and transcription factors, sites in adjacent coding sequences that form protein complex, or ncRNA that predominantly act in *cis*. As these interacting partners are often cotranscribed and translated in bacteria, we thus grouped co-selected pairs into *cis* and *trans* based on the length of transcription fragments reported in *B. pseudomallei* (Ooi et al. 2013). Here, pairs mapped to different genes or ncRNA but located within 7.68 kb (representing 95th percentile of polycistronic mRNAs) were termed *cis* pairs, whereas pairs located further than 7.68 kb apart or on separate chromosome were termed *trans* pairs. This resulted in 334 *cis* and 252 *trans* gene–gene pairs, 64 *cis* and 11 *trans* gene–ncRNA pairs, and 19 *cis* ncRNA–ncRNA pairs from the discovery data; of which 383 pairs (56.3%) were replicated in the validation data (fig. 1 and supplementary fig. S3 and table S3, Supplementary Material online). Despite *cis* pairs being more prevalent, the number of interacting partners per gene was fewer than the number of *trans* interacting partners (a mean of partners per *cis*-gene = 1.11, a mean of partners per *trans*-gene = 2.50) with the highest number observed in *BPSL1661* (*trans*-gene partners = 104 [discovery data], and 52 [validation data]).

### Moderate Functional Conservation of Genes under Co-selection

We assigned functional annotations to genes identified in both *cis* and *trans* gene–gene pairs. For both the discovery and validation data set, the majority of the co-selected genes were functionally assigned as cell envelope (21.9%), or transport/binding protein (10.6%), whereas many were left as uncharacterized or hypothetical proteins (26.7%) (supplementary fig. S4*A*, Supplementary Material online). We observed that a high proportion of the co-selected gene–gene pairs share the same functional annotations (51.3% of *cis* and 19.9% *trans* gene–gene pairs). To test whether this observation was driven by chance, we compared the number of pairs under the same or different functional annotations between 100 randomized and real data sets (see Materials and Methods). Excluding pairs with ambiguous annotations, functional conservation of *cis* gene–gene pairs for both discovery and validation data set were within the range random expectation (supplementary fig. S4*B*, Supplementary Material online). However, conservation of functional annotation of *trans* gene–gene pairs was greater than the random expectation for the discovery but not the validation data set (supplementary fig. S4*C*, Supplementary Material online). The inconsistency in results could be caused by a small sample size after an exclusion of genes with uncharacterized function. Nevertheless, our results indicate that co-selection of genes, at least in *trans* of the discovery data set, is driven by gene functions.

### Expression Patterns of Co-selected Gene–Gene Pairs

**Fig. 2.**
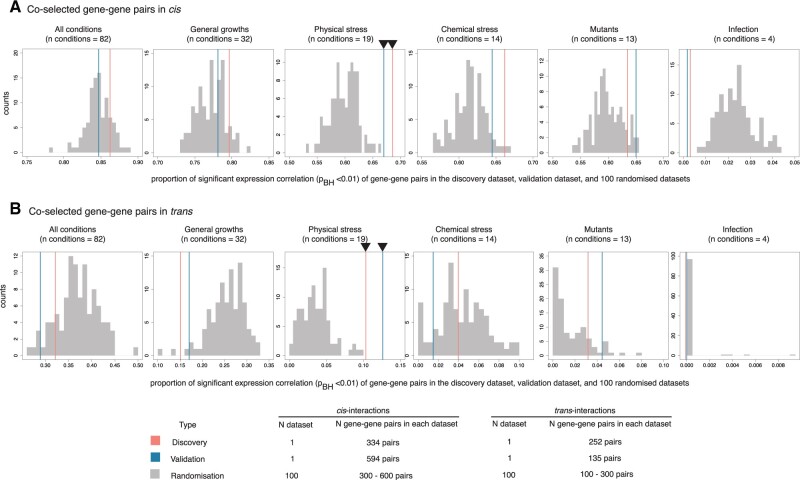
Expression correlation of co-selected gene–gene pairs. (*A* and *B*) Histograms represent the proportion of gene pairs with significant expression correlations for the *cis* and *trans* interactions, respectively. The expression conditions tested (from left to right) include all conditions (*n* = 82), general growth (*n* = 32), physical stress (*n* = 19), chemical stress (*n* = 14), mutants (*n* = 13), and infection (*n* = 4). For all plots, horizontal axis denotes the proportion of gene pairs with significant expression correlation (Benjamini–Hochberg adjusted *P* value <0.01) for the true co-selection signals found in discovery (red), validation (blue), and 100 randomized data sets (gray). Black triangles mark the conditions at which real genes under co-selection display greater proportion of significant expression correlation than 100 randomized controls.

We next sought to identify the conditions that might have driven the selective pressure resulted in observed patterns of co-selected genes. We used *B. pseudomallei* whole-genome tiling microarray expression data generated by Ooi et al. (2013), which assayed under a broad spectrum of conditions and exposure, including general growth (32 conditions), exposure to physicochemical stress (33 conditions), invasion assays (4 conditions), and defined genetic mutants (13 conditions) (supplementary fig. S1, Supplementary Material online). Here, genes expressed in ≥70 conditions are defined as being constitutively expressed as in Ooi et al. (2013). Approximately 22.4% of the genes that were detected in co-selection pairs in the discovery analysis or validation analysis, or both, were constitutively expressed compared with 39.5% of genes that were not part of any co-selection pairs (two-tailed Fisher’s exact test *P* = 5.74 × 10^−7^), indicating that co-selection signals were more strongly associated with condition-specific genes than those constitutively expressed. We next tested if the real data set showed a greater proportion of coexpressed genes compared with 100 randomized data sets (fig. 2). In addition, we examined under which conditions such coexpression patterns were observed. Using normalized gene expression profiles (Ooi et al. 2013), Pearson’s correlation analysis was performed for each gene–gene pair with transcription data from all conditions tested as well as from subsets of those conditions. Except for physical stress conditions, the proportion of coexpressed gene pairs from the real discovery and validation data sets either fell within or lower than the range of 100 randomized controls (significant expression correlation at Benjamini–Hochberg adjusted *P* value < 0.01). The results were consistent for *cis* and *trans* pairs, suggesting that physical stress has largely shaped the co-selection patterns at the time scale considered for this population. Here, physical stress conditions included temperature stress, osmotic stress, UV irradiation, and nutrient deprivation; the environmental conditions *B. pseudomallei* is regularly or seasonally exposed to (Limmathurotsakul et al. 2013; Baker et al. 2015; Ngamsang et al. 2015; Hantrakun et al. 2016; Manivanh et al. 2017; Bulterys et al. 2018). However, it should be cautioned that the results from *trans* gene–gene pairs were not completely independent but heavily driven by a co-selection hotspot, *BPSL1661*, which were linked to many other genes.

### Functional Characterization of Co-selection Hotspot

**Fig. 3.**
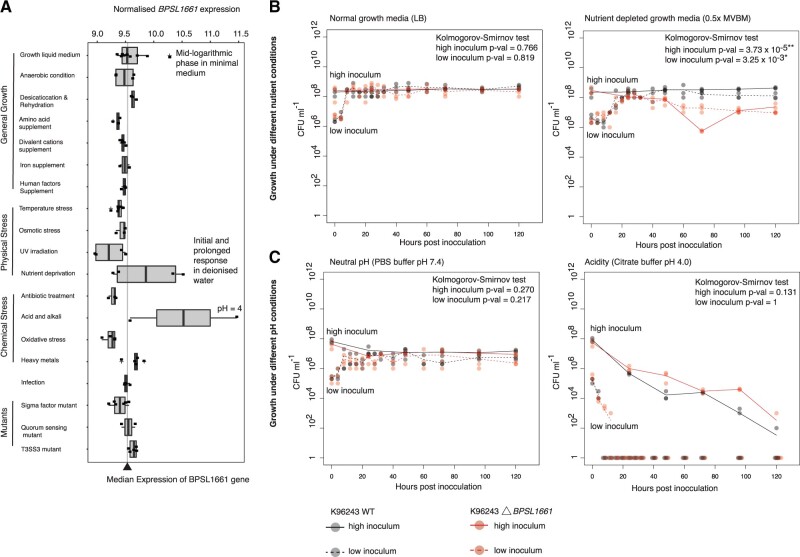
Functional characterization of *BPSL1661*. (*A*) Expression profile of *BPSL1661* across different conditions from Ooi *et al.* (2013), highlighting the gene upregulation during nutrient limited condition and high acidity. (*B*) and (*C*) represent growth- and stationary-phase survival of wild-type and *BPSL1661* knockout mutant from K96243 reference strain under changes in nutrition (*B*) and pH (*C*). Bacterial survival post inoculation at low (10^6^ CFU ml^−1^) and high (10^8^ CFU ml^−1^) concentration was determined as bacterial colony forming unit (CFU) at different time intervals. Three replicates were taken at each timepoint. Black and red dots denote observations from wild-type and *BPSL1661* mutant, respectively. Dotted and solid line represent profile of low and high bacterial inoculum, respectively. Difference in growth profiles between wild-type and mutant was measured using two-sided Kolmogorov–Smirnov test.

A putative adhesin gene *BPSL1661* was the hotspot of the largest co-selected gene network for both discovery and validation data set (fig. 1*B* and *D* and supplementary fig. S3, Supplementary Material online). *BPSL1661* codes for a secreted protein with a size ranging from 2,594 to 3,230 amino acids (approximately 325 kDa) in different isolates. The gene is located in a large highly variable genomic region termed genomic island 8 (GI8) (Tuanyok et al. 2008), proposed to be acquired through horizontal gene transfer (Holden et al. 2004). This genetic mobility possibly contributes to the presence of multiple *BPSL1661* alleles observed in our study. We detected six major alleles of *BPSL1661* (*n* ≥ 5 isolates, see Materials and Methods) consistent with previous reports on multiple protein epitopes (Suwannasaen et al. 2011; Kohler et al. 2016). All *BPSL1661* alleles share a conserved hemolysin-type calcium-binding domain which is common in proteins secreted through a type I secretion system, and two copies of the VCBS domain (a repeat domain found in *Vibrio*, *Colwellia*, *Bradyrhizobium*, and *Shewanella*) known to be involved in cell adhesion. Variations in the presence of bacterial Ig and flagellin domains in *BPSL1661* were observed in the study population. Interestingly, previous studies reported heterogeneity in human immune responses to polypeptides generated from different *BPSL1661* alleles, ranging from null to strong antibody responses (Felgner et al. 2009; Suwannasaen et al. 2011; Kohler et al. 2016). Such disparity in host recognition of different *BPSL1661* alleles potentially suggests that the protein may not principally function in host cell invasion but play other significant roles in *B. pseudomallei* survival. Transcription assays further revealed that *BPSL1661* is downregulated during infection but upregulated in acidic conditions (pH 4), mid-logarithmic phase in minimal media, and nutrient deprivation (fig. 3*A*); further indicating a role of *BPSL1661* in adaptation to environmental stress.

To better understand the function of *BPSL1661*, we knocked out the *BPSL1661* gene in the K96243 strain and compared the number of live cells of the mutant against the wildtype under the conditions *BPSL1661* was upregulated (fig. 3*B* and *C*), for 120 h. We observed no significant differences in bacterial survival in nutrient-rich growth media, neutral pH (pH 7.4), or acidic conditions (pH 4). This could be due to functional redundancy that compensates for the loss of a single gene function. However, the *BPSL1661* mutant showed a significant reduction in stationary-phase survival compared with the wildtype under nutrient-limited conditions (two-sided Kolmogorov–Smirnov test *P* value = 3.73 × 10^−5^ and 3.25 × 10^−3^ for high and low bacterial inoculum, respectively); confirming an essential role of *BPSL1661* under nutrient deprivation. Our observation of *BPSL1661* as a hotspot may imply that nutrient depletion is one of the major selective pressures underlying the co-selection patterns. This finding is also consistent with environmental sampling studies in Southeast Asia and Australia which have reported that the bacterium is commonly found in nutrient-depleted soils (Baker et al. 2015; Hantrakun et al. 2016).

The maintenance of different *BPSL1661* alleles in the population likely suggests that the gene has been under balancing selection. Although lower nutrient abundance appears to be a common feature across melioidosis endemic areas; the soil physiochemical properties, microbial diversity, temporal disturbances such as monsoon seasons and anthropogenic activities that alter the environmental conditions vary greatly (Kaestli et al. 2015; Musa et al. 2016; Ribolzi et al. 2016; Manivanh et al. 2017; Goodrick et al. 2018). These factors create patterns of spatial and temporal heterogeneity to which *B. pseudomallei* has adapted and possibly has led to the coexistence of multiple *BPSL1661* alleles detected in this study. We also noted geographical differences in *BPSL1661* allele frequencies. An allele harboring a flagellin domain (here denoted as allele A, supplementary fig. S5*A*, Supplementary Material online) was detected at lower frequency in Australia (28.7%), at moderate frequency in the Malay Peninsula (37.6% from Malaysia and Singapore) and higher frequency in the countries bordered by the great Mekong river (59.1% from Thailand, Laos, Cambodia, and Vietnam) (supplementary fig. S5*B*, Supplementary Material online). Such a difference in allele frequencies could be either driven by different local selection pressures, or by genetic drift (or both). Due to its horizontal mode of inheritance, an ancestral history of each *BPSL1661* allele could not be reliably reconstructed. Functional characterization of different *BPSL1661* alleles also warrants further future studies.

### *BPSL1661* Co-selection Network and Putative Bacterial Response under Low Nutrient Abundance

**Fig. 4.**
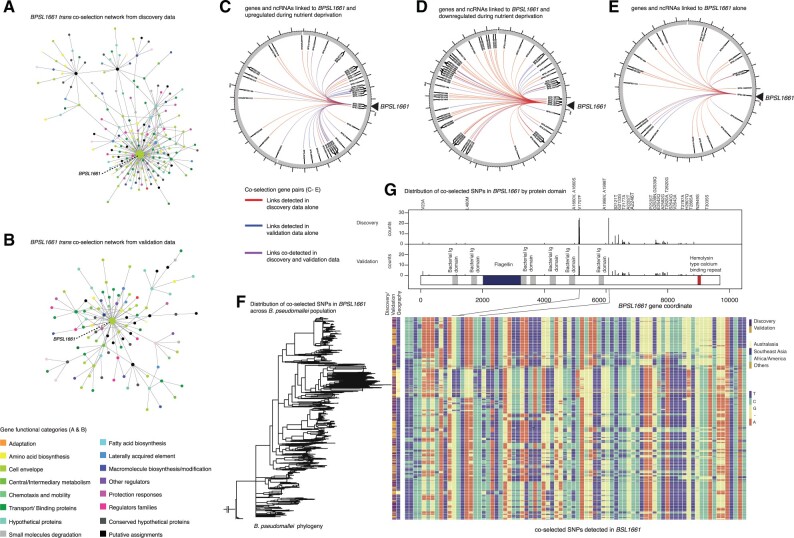
*BPSL1661* co-selected gene and ncRNA network. (*A*) and (*B*) represent networks of *trans* interacting co-selected gene–gene pairs in the discovery and validation data sets, respectively. Each node denotes a gene under co-selection with the node size proportional to numbers of pairs linked to the gene, and color coded by the gene functional category. For both discovery and validation data sets, *BPSL1661* consistently acts as hub of the *trans* co-selected gene network. (*C*), (*D*), and (*E*) summarize genes and ncRNA co-selected with *BPSL1661* in both discovery and validation data set: genes and ncRNA co-selected and upregulated under nutrient deprivation (*C*); genes and ncRNA co-selected but downregulated under nutrient deprivation (*D*); and genes and ncRNA that are co-selected alone (*E*). Links identified from the discovery data alone, validation data alone, and both data are colored as red, blue, and purple, respectively. (*F*) Distribution of *BPSL1661* SNPs (rows) across different population (columns). The estimated phylogeny is shown on the left with the first two columns labeled by type of data (discovery or validation data set) and geographical origins of isolates, respectively. The remaining columns demonstrate all nucleotide variants detected in *BPSL1661*. Variants coincide with peaks of co-selection signals were marked, one of which correspond to V1707F substitution.

We considered genes and ncRNA co-selected with *BPSL1661* in both the discovery (*n* gene pairs = 105, *n* ncRNA pairs = 5) and validation data set (*n* gene pairs = 53, *n* ncRNA pairs = 2) totaling 136 pairs, of which 29 pairs are shared in both data sets (fig. 4 and supplementary table S4, Supplementary Material online). The majority of these gene and ncRNA pairs were linked to *BPSL1661* in *trans* except for an outer membrane protein *BPSL1660* which paired in *cis*. During nutrient-depleted conditions (Ooi et al. 2013; Mohd-Padil et al. 2017), 43/129 of the *trans* gene pairs and 1/6 of ncRNA pairs were upregulated with *BPSL1661*, whereas 64/129 of the *trans* gene pairs and 2/6 of ncRNA pairs were downregulated, respectively. Many of these genes are predicted to encode proteins that participate in alternative metabolic pathways, energy conservation, uptake of external carbon source, cellular signaling, and transcriptional regulation (supplementary table S4, Supplementary Material online). A pyrophosphohydrolase (*spoT* or *BPSL2561*) is upregulated during nutrient starvation and is known to have a dual function to synthesize and hydrolyze guanosine tetra- and pentaphosphates (ppGpp) (Müller et al. 2012). In bacteria, ppGpp serves as a mediator in nutritional surveillance, coordinating a variety of cellular activities in response to changes in nutrient availability (Wang et al. 2007). In *B. pseudomallei*, deletion of ppGpp synthetase and hydrolase led to reduced survival during stationary phase compared with wildtype (Müller et al. 2012). It is possible that *B. pseudomallei* switches to alternative carbon sources to maintain cellular energy when complex sources such as glucose are not available. Genes encoding a C4-carboxylate transport transcription regulation protein (*BPSL0427*), a malate synthase (*BPSL2192*), and a glycogen branching enzyme (*BPSL2076*) were co-selected with *BPSL1661* but had different expression profiles under nutrient-limited conditions. A transport transcription regulation protein (*BPSL0427*) was upregulated under nutrient deprivation. Its homolog was shown to facilitate the cellular uptake of four carbon compounds such as aspartate, fumarate, and succinate when common carbon sources such as glucose are scarce (Wösten et al. 2017). Another upregulated gene, malate synthase (*BPSL2192*) is a key enzyme involved in the glyoxylate cycle and was also shown to be essential to *Mycobacterium tuberculosis* survival under nutrient starvation (Puckett et al. 2017). Its expression allows cells to utilize two carbon compounds to sustain carbon requirement in the absence of glucose. On the contrary, a glycogen branching enzyme (*BPSL2076*) was co-selected with *BPSL1661* but downregulated during nutrient depletion. A homolog of glycogen branching enzyme is known to facilitate glucose conversion into long-term glycogen storage when there are excessive carbon sources (Wang et al. 2019). The co-selection of *spoT*, *BPSL0427*, *BPSL2192*, and *BPSL2076* with *BPSL1661* may reflect the energy balance of cells under ranges of nutritional conditions through their evolutionary timeline. We also observed many ncRNA, transcription regulators, and DNA-binding proteins co-selected with *BPSL1661*. These genes and ncRNA may further regulate downstream responses under changes in nutrient abundance. Together, the *BPSL1661* co-selection network seems to suggest that *B. pseudomallei* has adapted to survive nutrient-limited conditions and/or hostile environments.

## Conclusion

This study is the first, in our knowledge, to deploy an integrated approach of GWES, transcriptomic analyses and knockout assays to understand the evolution and unique selective pressures acting on a microorganism. Although GWES detected signals for co-selected loci, transcriptomic data provided condition-dependent information on which selection pressures may have acted upon the detected loci. Nevertheless, our study has some limitations. First, our GWES focused only on nucleotide polymorphisms found by comparison to the *B. pseudomallei* K96243 reference genome. Co-selected loci on other types of structural variants including indels, genomic inversions, gene duplication, or horizontally acquired genes absent in K96342 will be missed from this analysis. Second, the condition-wide transcriptomic data employed in this study was generated by a microarray platform. A change in the resolution from nucleotide polymorphisms employed in GWES to a gene level used in microarray has led to a loss of signals as only SNPs mapped to genes (69.5%) could be interpreted by gene expression analysis. Because multiple SNPs could be mapped to a single gene, it is possible that each of these SNPs could lead to different product of gene expression. This information is not available with microarray platform. It is also possible that other transcriptional conditions the bacterium is exposed to in its native niche are missing. Together, the incomplete genomic and transcriptomic data warrant further studies to cover more complex genomic variants, and broader transcriptional conditions with higher genetic resolution.

Despite limitations, our integration of data offers stringent predictions on which genes are key to *B. pseudomallei* survival under specific conditions. In particular, the putative adhesin *BPSL1661* was identified as a hotspot of the co-selection map. Our gene knockout experiment confirmed that the gene is essential for survival under nutrient deprivation. This is consistent with the soil conditions in which *B. pseudomallei* are commonly found and provides an evolutionary evidence that *B. pseudomallei* has been adapted to nutrient-depleted environments. It is possible that different *BPSL1661* alleles may facilitate bacterial adherence to different surfaces, cells, or hosts. This attachment could lead to biofilm formation or the bacterium being internalized by host cells, both of which are known bacterial strategies to withstand nutrient deprivation (Brown et al. 2008; Petrova and Sauer 2012; Chomkatekaew et al. 2020). Our study has a strong implication that the presence of *B. pseudomallei* in nutrient-depleted soil may define geographical regions where humans are at risk of melioidosis. It is thus necessary to improve environmental health to assist melioidosis prevention.

## Materials and Methods

### Study Design

We conducted three complementary tests: a co-selection analysis to scan for any SNP–SNP pairs that are mutually detected more frequently than expected as a result of selection pressures; a condition-wide transcriptome analysis to identify conditions under which co-selected gene–gene pairs likely operate; and a gene knockout assay to confirm the function of a hotspot for co-selection signals.

### Whole-Genome Sequencing Collections, Mapping, and Annotation

We sought *B. pseudomallei* whole-genome sequences from the public database (Holden et al. 2004; Hayden et al. 2012; Price et al. 2013; Sahl et al. 2013; Daligault et al. 2014; Bugrysheva et al. 2015; Chen et al. 2015; Hsueh et al. 2015; Johnson, Baker, et al. 2015; Johnson, Bishop-Lilly, et al. 2015; McRobb et al. 2015; Nandi et al. 2015; Sidjabat et al. 2015; Song et al. 2015; Spring-Pearson et al. 2015; Viberg et al. 2015; Chapple et al. 2016; Price et al. 2016; Chewapreecha et al. 2017; Chewapreecha et al. 2019) and combined these with newly sequenced genomes totaling 2,011, and further divided them into a discovery data set (1,136 isolate genomes) and a validation data set (875 isolate genomes) (supplementary fig. S1*A* and *B*, Supplementary Material online). Their accession numbers are tabulated in supplementary table S1, Supplementary Material online. The discovery data set came from an older collection (1935–2013), whereas the validation data set represented a recently sequenced collection (1976–2018). The two data sets overlap geographically, spanning two major melioidosis endemic regions of Southeast Asia and Australia. These isolates came from environmental, animal, and human sources with the latter constituted the larger proportion due to availability of microbiology laboratories embedded in the clinical settings. However, over 91% of human cases represent recent acquisition from the environmental sources (Currie et al. 2000; Wiersinga et al. 2012, 2018), thereby reducing the chance of coevolutionary signals being shaped by human infection alone. For newly sequenced data, DNA libraries were sequenced on an Illumina Hiseq2000 with 100-cycle paired-end runs. Short reads were mapped to the reference *B.pseudomallei* genome K96243 using SMALT 0.7.6 (Ponstingl and Ning 2010). The SNPs were called as in Chewapreecha et al. (2017). For the discovery data set (*n* = 1,136), the alignment contained 389,476 SNPs, of which 206,019 and 183,457 were located in chromosomes I and II, respectively. For the validation data set (*n* = 875), the alignment contained 285,543 SNPs, of which 150,499 and 135,044 SNPs were located in chromosomes I and II, respectively. The genome K96243 was reannotated using curated genes, ncRNA, and functional predictions obtained from transcriptional assays described in Ooi et al. (2013) and Mohd-Padil et al. (2017) (supplementary fig. S1C and *D*, Supplementary Material online).

### Co-selection Tests

Co-selection analysis was separately performed on the sequence alignment of discovery and validation data sets using the mutual information-based GWES tool SpydrPick (Pensar et al. 2019) (supplementary table S2, Supplementary Material online). SNPs with minor allele frequency greater than 1% and gap frequency smaller than 15% were included in the analysis. To adjust for the population structure, sequence reweighting was applied using the default similarity threshold of 0.10. Direct links for which the mutual information exceeded the extreme outlier threshold, also after removing the influence of gaps, were selected for further examination. To discriminate signals influenced by the LD structure from the co-selection signals, SNP–SNP pairs were further categorized into *cis* or *trans* interaction based on the length of transcription fragments reported in *B. pseudomallei* (Ooi et al. 2013). Any pairs located on different genes or ncRNA but within 7.68 kb (representing 95th percentile of polycistronic mRNAs) were termed *cis* pairs, whereas pairs located further than 7.68 kb apart or on separate chromosome were termed *trans* pairs (fig. 1). These SNPs were mapped to genes and ncRNA from the curated *B. pseudomallei* reference genome K96243, resulting in *cis* or *trans* co-selection of gene–gene, gene–ncRNA, and ncRNA–ncRNA pairs (supplementary table S3 and S4, Supplementary Material online). SNP–SNP pairs located within the same gene, or ncRNA were removed for this analysis.

### Generation of Randomized Gene–Gene Cohorts

A hundred randomized data sets were separately generated for *cis* and *trans* interactions by randomly pooling any pairs of genes from the reference *B.pseudomallei* genome K96243. Here, *cis* randomized data sets were created from pairs located on the same chromosome and <7.68 kb apart, whereas *trans* randomized data sets were based on genes located >7.68 kb apart or on a different chromosome. The size of each randomized data set matched the size of real discovery and validation data set. For *cis* gene–gene interactions (*n* discovery = 334 pairs, *n* validation = 594 pairs), the sample size of 100 randomized controls ranged between 300 and 600 pairs. For *trans* gene–gene interactions (*n* discovery = 252 pairs, *n* validation = 135 pairs), the number of pairs in the randomized controls ranged between 100 and 300 pairs. This allowed us to generate a distribution of random expectation, which can be used to test the hypotheses for functional conservation and elevated expression correlation of genes under co-selection.

### Functional Classification of Genes under Co-selection

Different gene functional categories including a curated Riley’s classification, clusters of orthologous groups, KEGG Pathway (Kanehisa and Sato 2020), and gene ontology (GO terms) (The Gene Ontology Consortium 2019) were assigned to each gene–gene pair. The final analysis was focused on a curated Riley’s classification as it covered more genes detected for co-selection than other classification systems. For both discovery and validation data sets, we searched for enrichment of gene functions among co-selected genes against their distribution in K96243 genome using two-sided Fisher’s exact test (R function fisher.test()) while controlling for false positive from multiple testing using Benjamini–Hochberg method (Benjamini and Hochberg 1995).

To test whether genes under co-selection display functional conservation, we measured the proportion of gene pairs that shared the same functional annotation in the real data sets and compared the observations to the random expectation generated from 100 randomized controls (supplementary fig. S4, Supplementary Material online). Pairs comprising ambiguous gene annotations including uncharacterized or hypothetical proteins were removed from the analysis.

### Expression Patterns of Genes and ncRNA under Co-selection

We used *B. pseudomallei* condition-specific expression comprising 165 array profiles generated from Ooi et al. to elucidate the function of co-selected gene–gene pairs. The data had been log-transformed to fit a Gaussian distribution. Expression correlations between gene–gene pairs were defined by Pearson correlation using R function cor.test(gene1, gene2, method = “pearson”). We applied Benjamini–Hochberg adjusted correction for multiple testing. Expression profiles were categorized into five major categories spanning general growth, physical stresses, chemical stresses, infection, and mutant conditions. For all conditions, and each of five major expression condition; we compared the proportion of pairs with significant expression correlation (Benjamini–Hochberg adjusted *P* value < 0.01) from the real co-selected gene–gene pairs against the distribution of random expectation generated from 100 randomized controls (fig. 2). Expression profile of *BPSL1661* was obtained from Ooi et al. and cross-checked for consistency with (Rodrigues et al. 2006; Chin et al. 2015; Jitprasutwit et al. 2020) where conditions were overlapped.

Additional pattern of ncRNA expression under nutrient limitation was sought from Mohd-Padil et al. (2017). The authors measured and compared ncRNA expressed when *B. pseudomallei* was subjected to nutrient rich BHIB media and nutrient-depleted M9 condition.

### Genetic Variations in *BPSL1661* and Their Distribution across the Core Genome Phylogeny

Where complete genomes were available, BLAT v. 36 (Kent 2002) was used to locate the position of *BPSL1661* homolog and further confirmed with genome annotations. Illumina-sequenced short reads were assembled as in Chewapreecha et al. (2019) and annotated using Prokka v.1.14.5 (Seemann 2014). Coding sequence of *BPSL1661* was identified using BLAT and confirmed with gene annotation. *BPSL1661* sequences from all genomes were aligned using MAFFT v.7.407 (Katoh 2002) and assigned into different clusters using CD-HIT-EST v.4.8.1 (Fu et al. 2012) with sequence identity threshold of 0.9. Here, any clusters with ≥5 members were considered representative alleles, resulted in six *BPSL1661* alleles across the data sets. Protein domains of each *BPSL1661* allele were sought from CDD/SPARCLE v.3.17 conserved domain database (Lu et al. 2020) (supplementary fig. S5, Supplementary Material online).

To investigate the distribution of *BPSL1661* variants across *B. pseudomallei* population, core-genome phylogeny was constructed from core genome SNP alignment. SNPs were called from K96243 mapped genome alignment using SNP sites v.2.5.1 (Page et al. 2016) excluding sites associated with mobile genetic elements (Tuanyok et al. 2008). We next estimated a maximum-likelihood tree using IQ-TREE v.1.6.10 (Minh et al. 2020) using General Time Reversible + Gamma distribution model of nucleotide substitution with default heuristic search options and 1,000 bootstraps (Minh et al. 2013) (supplementary fig. S5, Supplementary Material online).

### *BPSL1661* Functional Characterization

The hub of co-selection signals, *BPSL1661* was next functionally characterized. Bacterial strains, plasmids, and oligonucleotides used in this study are listed in supplementary tables S5 and S6, Supplementary Material online. GF-1 bacterial gDNA extraction kit and deoxynucleotide triphosphates were purchased from Vivantis; Platinum DNA Taq polymerase from Invitrogen; pGEM-T Easy vector systems from Promega; KOD Plus DNA polymerase from Toyobo; restriction enzymes from New England BioLabs; QIAquick Gel Extraction kit, MinElute PCR purification kit from Qiagen; Ampicillin (Ap), Kanamycin (Km), and Gentamycin (Gm) were purchased from Sigma, and Isopropyl β-d-1-thiogalactopyranoside (IPTG), 5-bromo-4-chloro-3-indolyl-β-d-glactopyranoside (X-Gal), and 5-bromo-4-chloro-3-indolyl-β-d-glucuronide (X-Gluc) were purchased from Gold Biotechnology.

The culture of *B. pseudomallei* K96243 wild-type, *B. pseudomallei BPSL1661* clean mutant, and the *Escherichia coli* strains used for construction of the *B. pseudomallei* mutant were routinely grown in Luria-Bertani (LB) and Luria-Lennox (LB, low salt) medium (Sigma, USA), at 37°C with 200 rpm agitation. When necessary, the medium was supplemented with antibiotics, chemicals, and chromogens at the concentrations of 100 µg ml^−1^ Ap, 35 µg ml^−1 ^Km, 0.1 mM IPTG, 50 µg ml^−1^ X-Gal for *E. coli*; 5 µg ml^−1^ Gm, 1,000 µg ml^−1 ^Km, 0.1 mM IPTG, 50 µg ml^−1^ X-Gluc for *B. pseudomallei.*

### Construction of the *BPSL1661* Clean Deletion Mutant

The nucleotide sequence encoding (*BPS_RS08795* [*BPSL1661*]) gene (GenBank accession no. WP_045606470.1) of *B. pseudomallei* K96243 was used to design primers for clean deletion using Primer-BLAST program (Ye et al. 2012). The BPSL1661 clean mutant (Δ*BPSL1661*) (supplementary fig. S6, Supplementary Material online) was constructed from *B. pseudomallei* K96243 by double-crossover allelic exchange as described previously (López et al. 2009). Briefly, the upstream and downstream DNA fragments of *BPSL1661* were amplified using BPSL1661_PFup/PRup and BPSL1661_PFdown/PRdown primers and then ligated. The ligated amplicons were cloned into TA cloning vector pGEM-T Easy (Promega, USA) and check for its correct insert size before subcloned into an allelic exchange plasmid pEXKM5 (López et al. 2009). The recombinant pEXKM5 plasmids were introduced into *B. pseudomallei* K96243 by biparental conjugation using conjugal *E. coli* S17-llpir (Simon et al. 1983). Merodiploid was selected on LB medium supplemented with X-Gluc and Kanamycin that appeared as pale blue colonies. To obtain the clean deletion Δ*BPSL1661* mutant, merodiploid colonies were cultured in LB medium to reach stationary phase then subculture into YT broth supplemented with 10% sucrose for overnight and spread on LB medium with 15% sucrose. Suspected *B. pseudomallei* colonies was verified by PCR and confirmed by DNA sequencing of the region flanking *BPSL1661*.

### *BPSL1661* Condition-Specific Assays

Guided by expression profile of *BPSL1661* in Ooi et al., the growth of wild-type and Δ*BPSL1661* were compared by enumerating the bacterium grown under normal growth (LB broth), nutrient-depleted condition (modified Vogel and Bonner’s medium, MVBM), neutral pH, and acidic pH. The bacterium was grown on Ashdown’s selective agar (Ashdown 1979) before transferring to grow under each condition.

We followed Ooi et al. for normal growth condition using LB broth. We artificially induced nutrient-limited condition in vitro by replacing glucose-limited media (six carbon source) with glycerol (three carbon source) in MVBM. Moreover, MVBM (Lam et al. 1980) further forced cell starvation by inducing biofilm formation. Nutrients will be consumed by cells located on the periphery of the biofilm clusters leading to reduced level of nutrients diffused to the inner cells. Both wild-type and Δ*BPSL1661* were grown on 0.05% glucose in 0.5 × MVBM [0.05%G, 0.5×MVBM]) at 37 °C with 200 rpm shaking for overnight. The culture was adjusted and measured an optical density (OD) at OD 600 nm to be 0.2 (OD_600_ = 0.2) and then inoculated into 10 ml prewarmed 0.05% G, 0.5×MVBM medium at 37 °C with 100 rpm shaking.

Growth in neutral pH was conducted in 10-fold diluted phosphate-buffered saline (PBS) pH 7.4. Growth in a low pH was done as described previously (Inglis and Sagripanti 2006). For both condition, bacteria were cultured for overnight in LB broth at 37 °C with 200 rpm shaking and subcultured in new fresh medium until log phase. Bacterial cells were washed in 1×PBS buffer pH 7.4 or 1×citrate buffer pH 4.0 for three times by centrifugation (8000 × g for 5 min) and resuspended in 10 ml of each buffer. For all conditions, we observed bacterial initial growth and stationary-phase survival for both low (10^6^ CFU ml^−1^) and high (10^8^ CFU ml^−1^) inoculum. The colony forming unit (CFU) post inoculation was counted at different time interval. All assays involved three replicates that were independently prepared, cultured, and treated. Difference in growth profile between wild-type and Δ*BPSL1661* for each condition was compared by a nonparametric Kolmogorov–Smirnov test (Berger and Zhou 2014) (fig. 3). 

### Visualization

Visualization of phylogenetic trees was performed in Phandango (Hadfield et al. 2018). Figures were plotted using R (R Core Team 2017) and R applications (Yu et al. 2018).

### Statistics and Reproducibility

No sample sizes were predetermined. For categorical data, two-sided Fisher’s exact tests were employed to compare differences between each group. For continuous data, nonparametric Mann–Whitney *U* tests and a parametric Pearson correlation were used to compare the intergroup distribution and the correlation of the expression levels between each co-selected gene–gene pair, respectively. Where appropriate, we used Benjamini–Hochberg procedure to adjust *P* value for multiple comparisons.

## Supplementary Material

Supplementary data are available at *Molecular Biology and Evolution* online.

## Supplementary Material

msab306_Supplementary_Data
