# Supplementary material for: Co-evolutionary Signals Identify Burkholderia pseudomallei Survival Strategies in a Hostile Environment
Source: Mol Biol Evol. 2021 Oct 18;39(1):msab306. doi: 10.1093/molbev/msab306 (PMC8760936; doi:10.1093/molbev/msab306)
Supplement: msab306_Supplementary_Data [file msab306_supplementary_data.zip › Chewapreecha.Supplementary.Figure.tables.pdf]

Supplementary Information for

Co-evolutionary signals identify *Burkholderia pseudomallei* survival strategies in a hostile environment

Claire Chewapreecha, Johan Pensar, Supaksorn Chattagul, Maiju Pesonen, Apiwat Sangphukieo, Phumrapee Boonklang, Chotima Potisap, Sirikamon Koosakulnirand, Edward J Feil, Susanna Dunachie, Narisara Chantratita, Direk Limmathurotsakul, Sharon J Peacock, Nick PJ Day, Julian Parkhill, Nicholas R Thomson, Rasana W Sermswan, Jukka Corander

This file contains **Figure S1 – S6**, legends for **Table S1- S4** (provided separately as Excel Spreadsheet or text format), and **Table S5 - S6**.

Figure S1

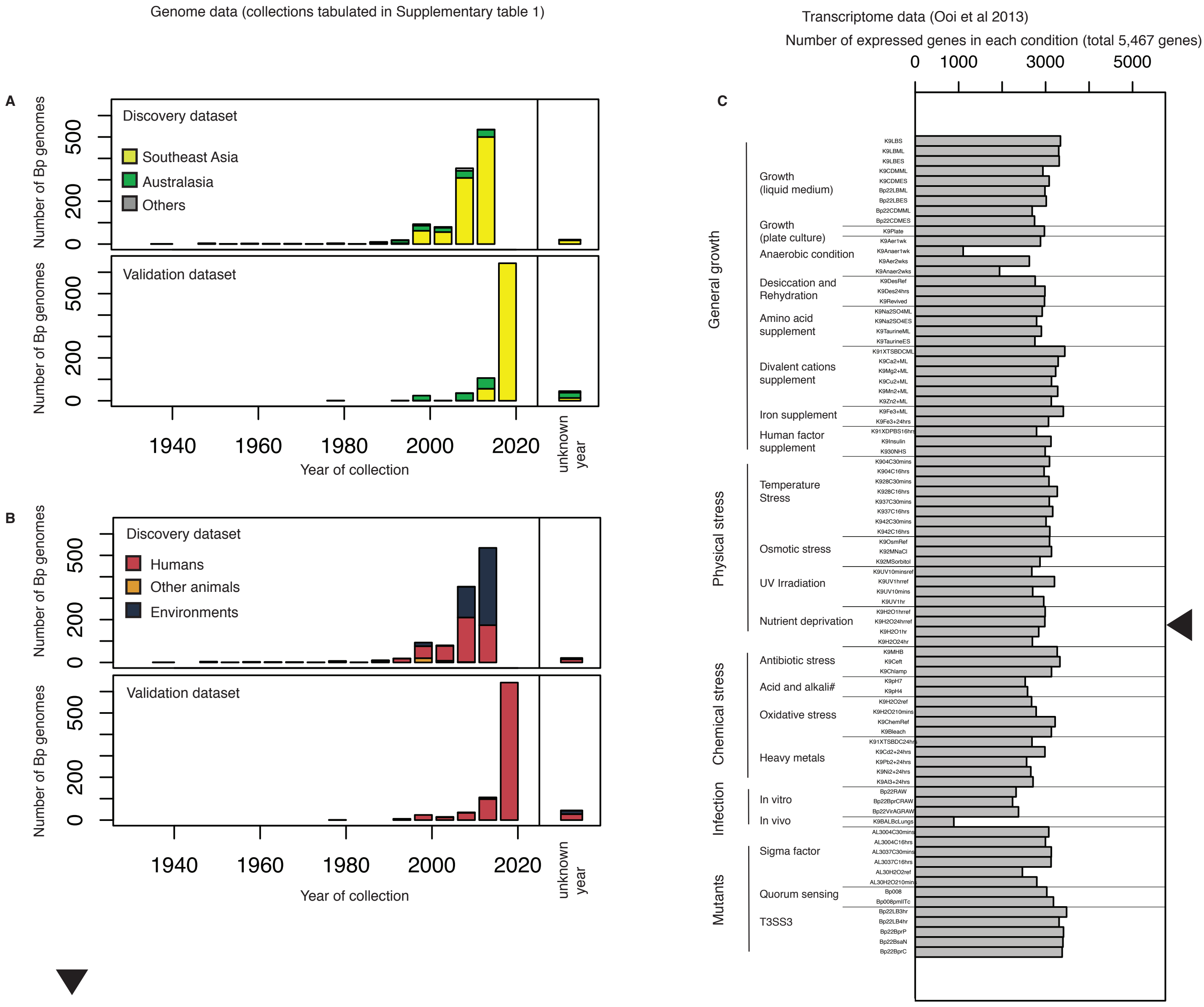

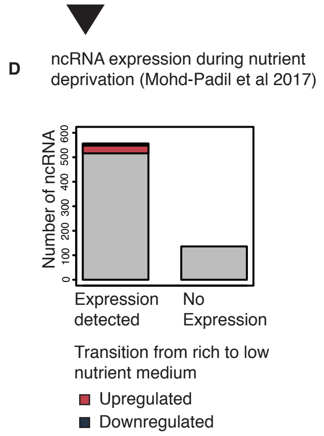

Figure S1 Summary of genomic and transcriptomic data used for the analyses. A) - B) represent distribution of *B. pseudomallei* genomes in the discovery and validation dataset by year of collection with geographical origins and source of isolates plotted in A) and B) respectively. C) summarises *B. pseudomallei* expression profile across 82 conditions covering general growth, physical stress, chemical stress, infection and mutants obtained from from Ooi *et al.* The bar chart highlights the number of genes expressed in each condition. D) represents the expression profile of *B. pseudomallei* non-coding RNA in nutrient-rich and nutrient-limited conditions. The data was obtained from Modh-Padil *et al.*

Figure S2

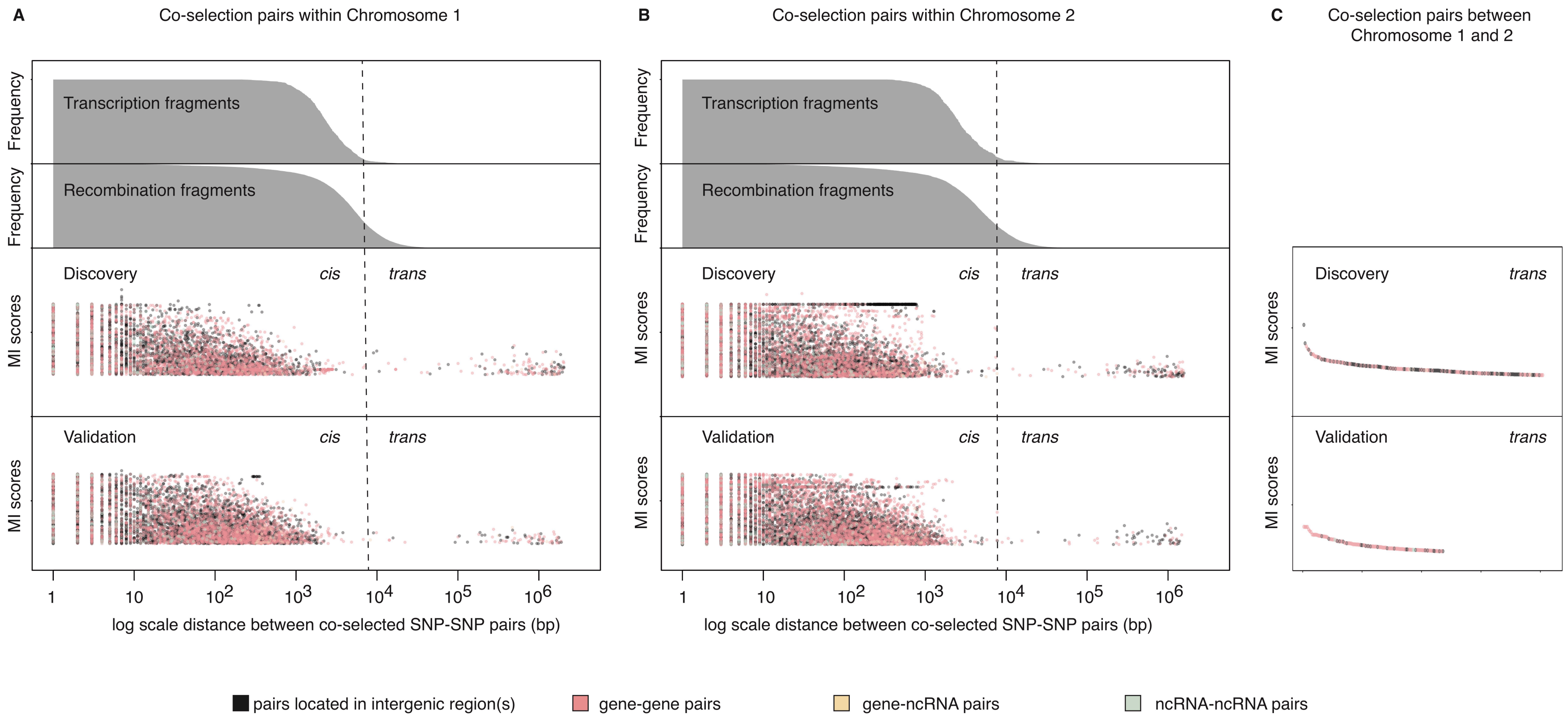

Figure S2 Co-selected SNP-SNP pairs.
A) - B) represent co-selection sites detected on Chromosome 1, Chromosome 2, and between Chromosome 1 and 2, respectively. For A) & B) The horizontal axis denotes physical distance on the chromosome on the log10 scale. The top to bottom panels represent the accumulated size of transcription fragments, the accumulated size of recombination fragments, the distance between co-selected SNP-SNP pairs against the mutual information scores from the discovery datasets, and the validation datasets, respectively. For two bottom panels, each dot represents a co-selected SNP-SNP pair and is colour-coded by their associations with genes (pink), a gene and a molecule of non-coding RNA (orange), molecules of non-coding RNA (green), or other intergenic regions (black). Vertical dotted lines denote 95th percentile of transcription fragments.
C) Top to bottom panel represent the mutual information scores of each co-selected SNP-SNP pair identified in the discovery and validation datasets, respectively. The same colour scheme was employed as in A) & B).

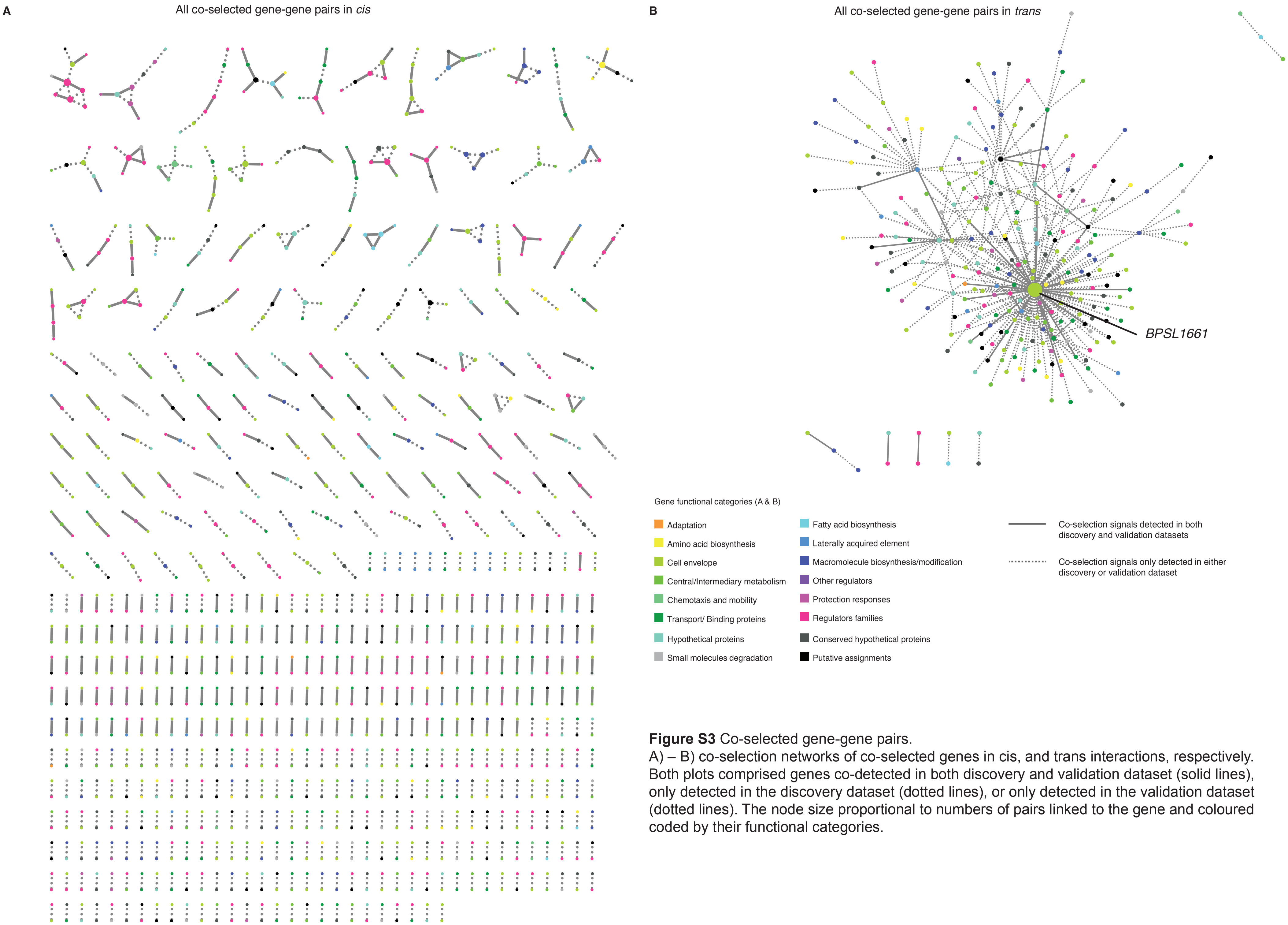

Figure S3 Co-selected gene-gene pairs.
A) – B) co-selection networks of co-selected genes in cis, and trans interactions, respectively. Both plots comprised genes co-detected in both discovery and validation dataset (solid lines), only detected in the discovery dataset (dotted lines), or only detected in the validation dataset (dotted lines). The node size proportional to numbers of pairs linked to the gene and coloured coded by their functional categories.

Figure S4

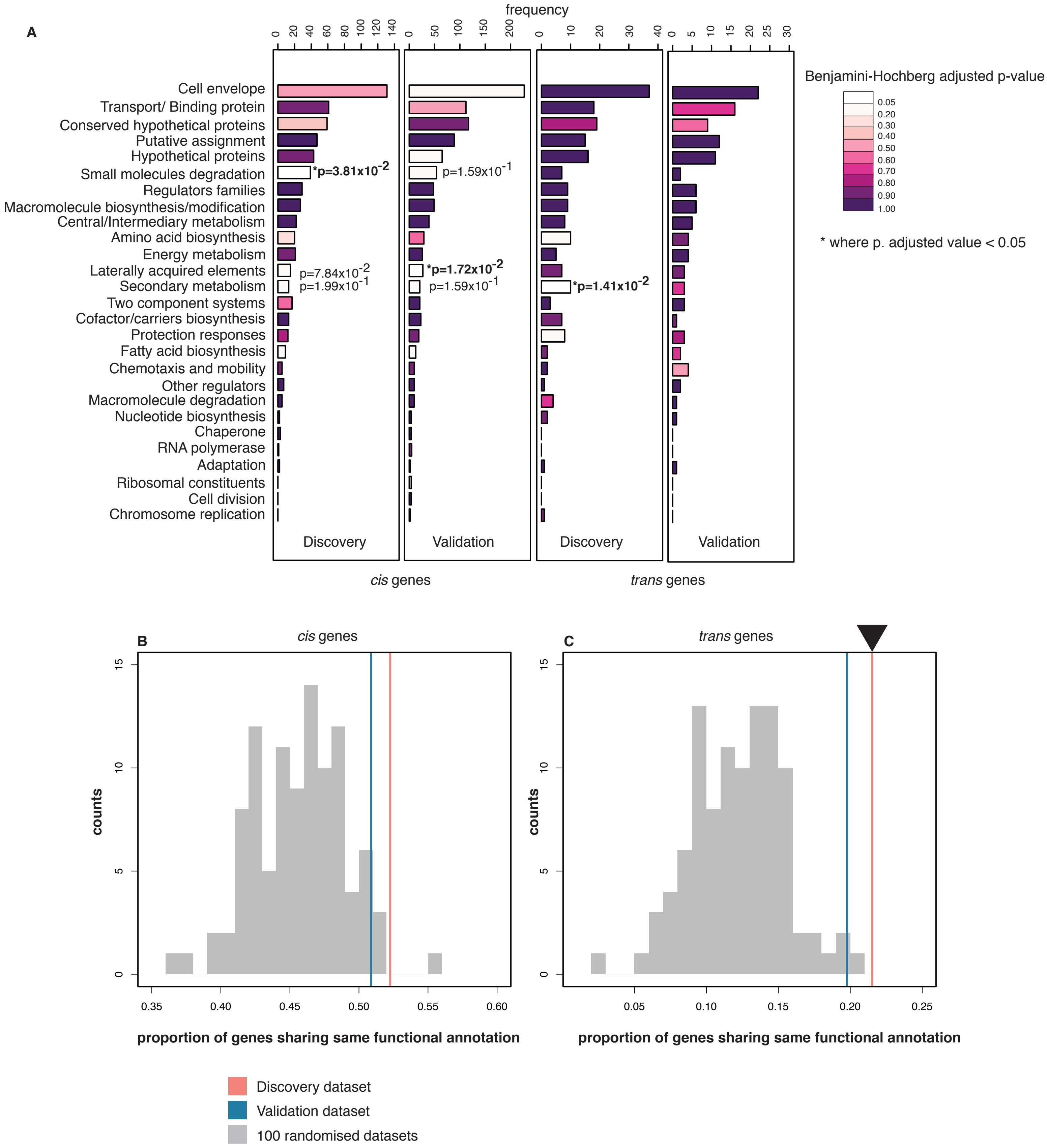

Figure S4 Functional category of co-selected genes.
A) Enrichment of functional category observed for *cis*- and *trans*- interactions in both discovery and validation datasets. The colour indicates the significance of the enrichment test result, ranging from white (adjusted p-value <0.05) to dark purple (adjusted p-value =1). Although there were moderate enrichments of terms associated with secondary metabolism, laterally acquired elements, and small molecule degradation in genes under co-selection (Benjamini-Hochberg adjusted p-value = 1.41 x 10-2, 1.72 x 10-2 and 3.81 x 10-2, respectively), none of these reached significance across both the discovery and validation datasets. B) & C) represent proportion of co-selected gene-gene pairs with same functional categories observed for *cis*- (B) and *trans*- (C) interactions. Data obtained from the discovery, validation, randomised dataset were highlighted as red, blue, and grey, respectively. The proportion of pairs with same functional categories were compared against the random expectation generated from 100 randomised controls. Pairs with ambiguous annotations comprising terms “conserved hypothetical/hypothetical proteins” or “putative assignment” were excluded from the analyses. Black triangle marks the dataset at which real genes under co-selection display greater proportion of pairs sharing the same functional annotation than 100 randomised controls.

Figure S5

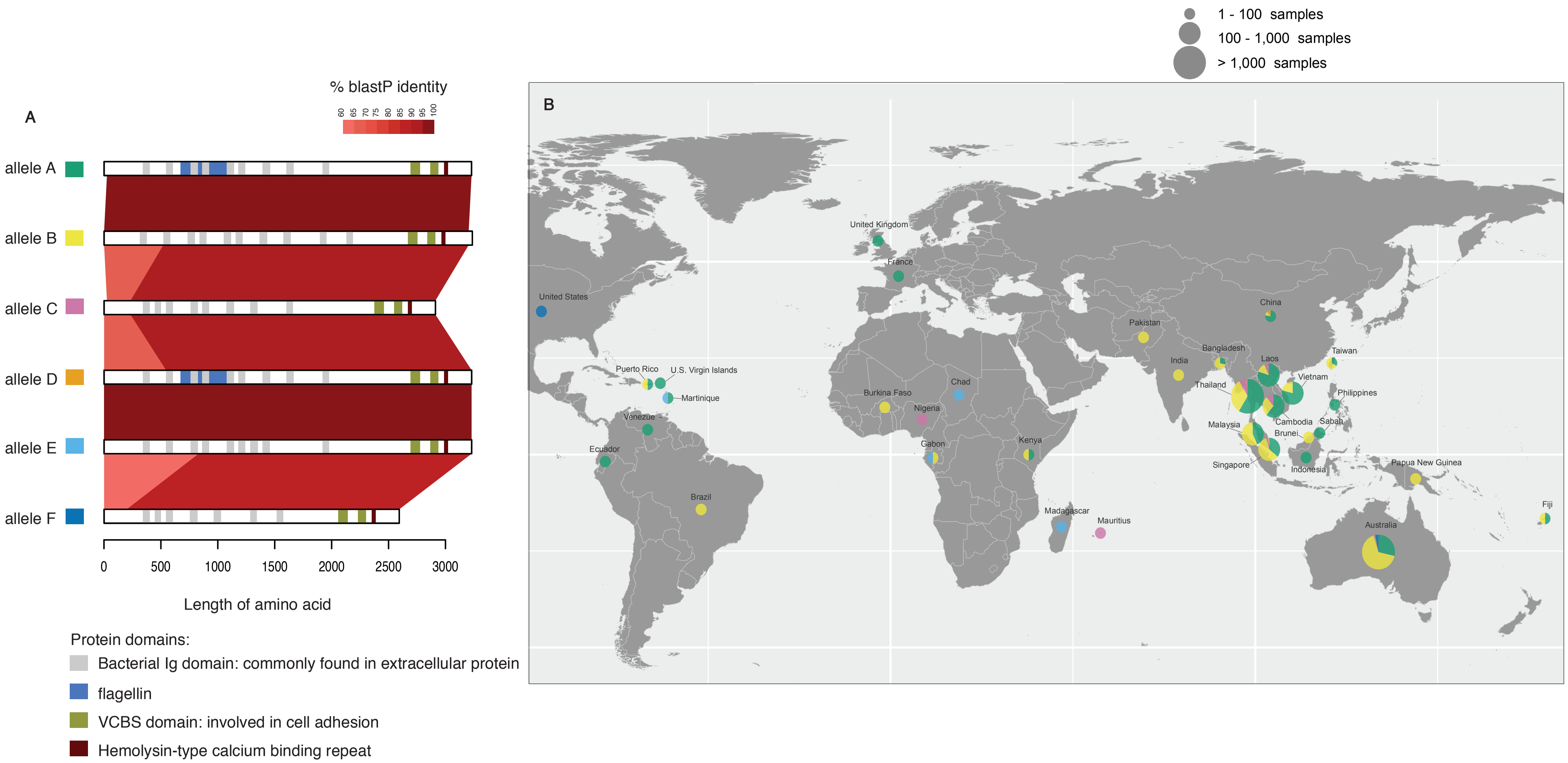

Figure S5 Distinct *BPSL1661* alleles and their geographical distribution.
A) represents diagram of six different *BPSL1661* alleles (denoted A to F). Each allele harbours different combination of protein domains including bacterial Ig domain (grey); flagellin (blue); *Vibrio, Colwellia, Bradyrhizobium*, and *Shewanella* domain (VCBS, green); and hemolysin-type calcium binding repeat (brown). Red areas show protein identity.
B) a world map shows geographical distribution of different *BPSL1661* alleles. Colour in pie charts correspond to different *BPSL1661* alleles, with the size of the pie charts proportional to number of samples obtained from each area

Figure S6

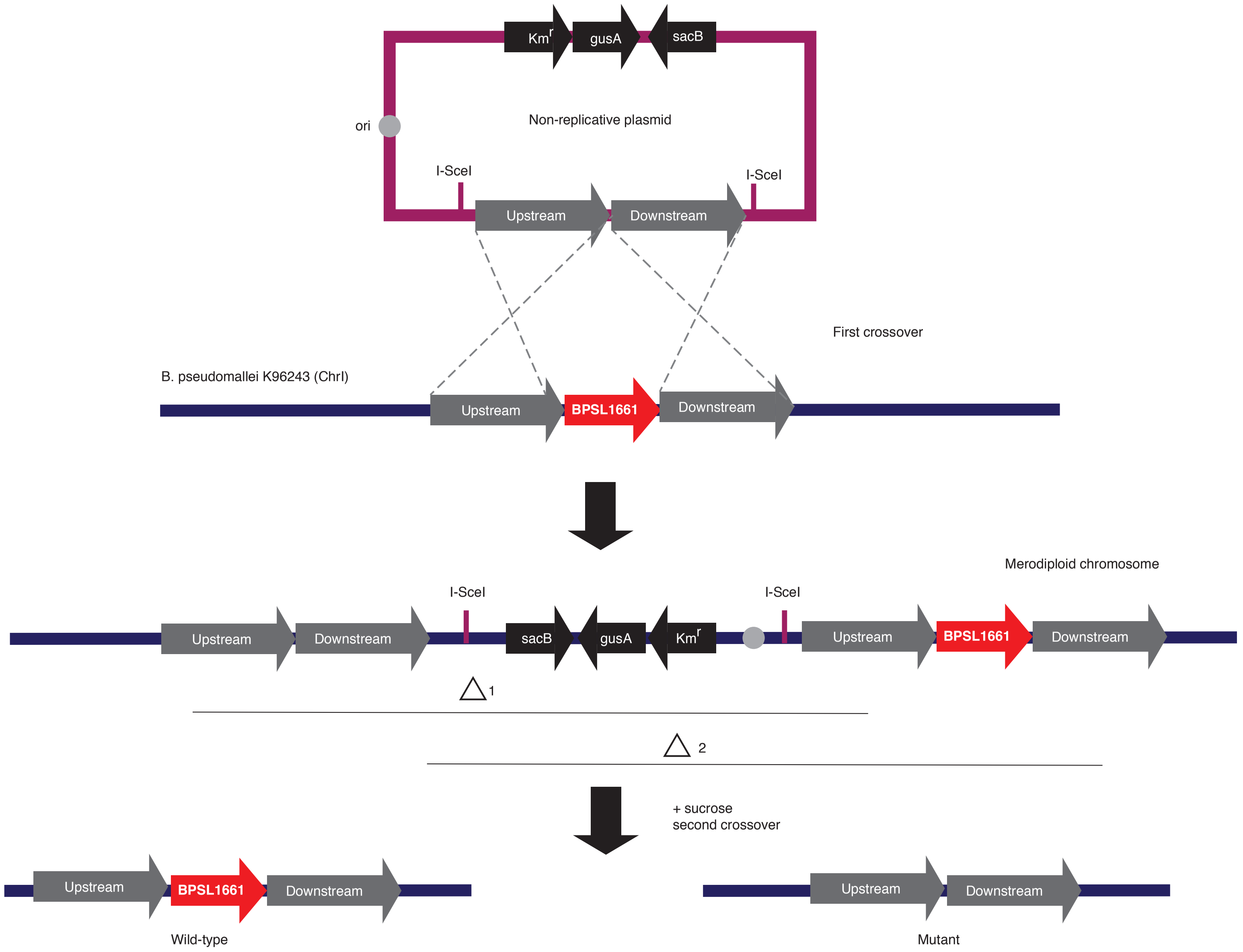

Figure S6 Construction of *BPSL1661* mutant.
Schematic diagrams of plasmid-based allelic exchange of *B. pseudomallei* K96243 to construct *BPSL166*1 mutant as modified from Lopez *et al.* 200950. A crossed PCR amplicon was generated from the assembled segments containing the upstream and downstream sequence of *BPSL1661* and cloned into pEXKM5, to construct the recombinant pEXKM5Δ*BPSL1661*;Kmr. (b) The recombinant plasmids were integrated into chromosome of *B. pseudomalle*i by homologous recombination (dash line denoted as the 1st crossover). The integrated recombinant plasmid replaced the *BPSL166*1 in the *B. pseudomallei* K96243 chromosome, called merodiploid. The sucrose counter selection was used to select for double crossovers (2nd crossover), resulting in generation of either in a wild type strain (Δ1) or *BPSL1661* mutant strain (Δ2).

Table S1 Isolate and accession codes for both short reads and assemblies used in the discovery and validation cohorts.

(The file can also be accessed through https://figshare.com/s/ed75b8c097a82a6fadd9)

Table S2 A summary of co-selected SNP-SNP sites and their mutual information scores detected in both discovery and validation datasets, in discovery dataset alone, or in validation datasets alone. The coordinates of the SNPs are based on *B. pseudomallei* K96243 reference. (The file can also be accessed through https://figshare.com/s/2af9a8ae37809af0a802)

Table S3 A summary of all co-selected gene-gene pairs, gene-ncRNA pairs and ncRNA-ncRNA detected in both discovery and validation datasets, in discovery dataset alone, or in validation dataset alone.

(The file can also be accessed through https://figshare.com/s/5686253fe1d19e79f639)

Table S4 A summary of genes (spreadsheet 1) and non-coding RNA (spreadsheet 2) co-selected with *BPSL1661* and their expression profile under different nutrient conditions.
(The file can also be accessed through https://figshare.com/s/8b9e9f08d05e9045ebc8)

Table S5

Bacterial strains and plasmids used in this study

	**Bacterial strains & plasmids**	**Description**	**Source of Reference**
Bacteria	*B. pseudomallei* K96243	Clinical isolate from a patient admitted to Khon Kaen provincial hospital, Khon Kaen, Thailand	(Holden *et al*., 2004)
	B. pseudomallei K96243 [*BPSL1661* clean mutant]	K96243 derivative; Δ *BPSL1661*	This study
	E. coli DH5α	General cloning	(Edgar *et al*., 2002)
	E. coli S17-llpir	Mobilizing strain, S17-1 with a l prophage carrying the gene encoding the π protein; Cm^s Tc^s Gm^s Km^s Tp^r Px^s Sm^r	(Simon *et al*., 1983)
Plasmids	pGEM-T Easy	3,015-bp TA vector; pUC/M13; Ap^r	Promega
	pGEM-T Easy Δ*BPSL1661*	pGEM-T Easy::Δ*BPSL1661 B. pseudomallei* K96243; Ap^r	This study
	pEXKM5	Allelic exchange plasmid; Km^r	(Lopez et al., 2009)
	pEXKM5 Δ*BPSL1661*	pEXKM5::Δ*BPSL1661 B. pseudomallei* K96243; Km^r	This study

Ap, ampicillin; Km, kanamycin; r = resistance; s = sensitive

Table S6

Primers used in this study (GenBank accession number:WP_045606470.1)

	Primer name	Primer sequence [5' to 3']	Amplicon size (bp)	Reference or source
Deletion	BPSL1661_PFup<br>BPSL1661_PRup	TGCCA**GAATTC**CGAAACATCCATATTGCA<br>**CGCTCATTGTCGACTTTG**TTGCAATTCTCCTATTCG	787	This study
	BPSL1661_PFdown<br>BPSL1661_PRdown	**CGAATAGGAGAATTGCAA**CAAAGTCGACAATGAGCG<br>TATAA**CCCGGG**GCTGGTGATGGTGGCGTGAA	677	This study
Confirmation	BPSL1661_PFup<br>BPSL1661_PRdown 2	TGCCA**GAATTC**CGAAACATCCATATTGCA<br>CGAGCAGCACGGAAAGAAGCG	631	This study
